# Expression and Purification of Recombinant Hemoglobin in *Escherichia coli*

**DOI:** 10.1371/journal.pone.0020176

**Published:** 2011-05-20

**Authors:** Chandrasekhar Natarajan, Xiaoben Jiang, Angela Fago, Roy E. Weber, Hideaki Moriyama, Jay F. Storz

**Affiliations:** 1 School of Biological Sciences, University of Nebraska, Lincoln, Nebraska, United States of America; 2 Zoophysiology, Department of Biological Sciences, Aarhus University, Aarhus, Denmark; University of York, United Kingdom

## Abstract

**Background:**

Recombinant DNA technologies have played a pivotal role in the elucidation of structure-function relationships in hemoglobin (Hb) and other globin proteins. Here we describe the development of a plasmid expression system to synthesize recombinant Hbs in *Escherichia coli*, and we describe a protocol for expressing Hbs with low intrinsic solubilities. Since the α- and β-chain Hbs of different species span a broad range of solubilities, experimental protocols that have been optimized for expressing recombinant human HbA may often prove unsuitable for the recombinant expression of wildtype and mutant Hbs of other species.

**Methodology/Principal Findings:**

As a test case for our expression system, we produced recombinant Hbs of the deer mouse (*Peromyscus maniculatus*), a species that has been the subject of research on mechanisms of Hb adaptation to hypoxia. By experimentally assessing the combined effects of induction temperature, induction time and *E. coli* expression strain on the solubility of recombinant deer mouse Hbs, we identified combinations of expression conditions that greatly enhanced the yield of recombinant protein and which also increased the efficiency of post-translational modifications.

**Conclusion/Significance:**

Our protocol should prove useful for the experimental study of recombinant Hbs in many non-human animals. One of the chief advantages of our protocol is that we can express soluble recombinant Hb without co-expressing molecular chaperones, and without the need for additional reconstitution or heme-incorporation steps. Moreover, our plasmid construct contains a combination of unique restriction sites that allows us to produce recombinant Hbs with different α- and β-chain subunit combinations by means of cassette mutagenesis.

## Introduction

Hemoglobin (Hb) and myoglobin (Mb) have long held center stage in studies of the relationship between protein structure and protein function. Hb and Mb were the first proteins whose structures were solved at high resolution by X-ray crystallography, and Hb has served as a highly profitable model system for establishing principles of allosteric regulation [1], [2], [3], [4]. In the last few decades, our understanding of structure-function relationships has been greatly aided by the ability to introduce specific mutations into recombinant proteins by site-directed mutagenesis [5], [6], [7], [8], [9]. In the case of human embryonic Hbs, our understanding of structure-function relationships is solely based on experimental studies of recombinant proteins [10]. The expression and functional analysis of recombinant Hbs (rHbs) has also provided detailed insights into molecular mechanisms of pathophysiology and biochemical adaptation. For example, site-directed mutagenesis of human rHbs revealed the molecular mechanism responsible for elevated Hb-O_2_ affinity in birds that are capable of high-altitude flight [11], [12]. Similarly, mutagenesis studies of human rHbs also aided the identification of sites responsible for the binding of bicarbonate ions as allosteric effectors in crocodile Hb [13]. By using ancient DNA as source material, this experimental approach has even provided insights into functional properties of Hb from extinct animals [14].

Recent advances in molecular biology have led to the development of increasingly refined and efficient methods for producing rHb. Some of these methodological improvements have been motivated by the goal of developing cell-free, rHb-based blood substitutes [15]. Nagai and Thøgersen [16], [17] developed the first bacterial expression system for producing rHbs in *E. coli*. These first efforts involved fusing α- or β-globin cDNAs to the coding region of a bacteriophage repressor gene. The insoluble fusion protein could then be digested to recover the intact globin chain [18]. One drawback of this system was that, once recovered, the globins had to be reconstituted *in vitro* with heme. A second-generation expression system involved co-expressing the α- or β-globin genes as a polycistronic transcript with *tac* promoter [19]. In this system, functional Hb tetramers were produced after the incorporation of exogenous heme in the *E. coli* cytoplasm. One problem with this method was the lack of N-terminal processing, such that the N-terminal methionine residue was not cleaved from the α- and β-chain polypeptides. Two strategies have been adopted to solve this problem [19]. One strategy involves altering the first codon so that valine is substituted for the initiator methionine. The second strategy involves co-expressing the gene for methionine aminopeptidase (MAP), an enzyme that cleaves the N-terminal methionine from the nascent globin chains in *E. coli* [20], [21]. In the polycistronic approach the α- and β-globin genes were successfully co-expressed with the MAP gene under the control of two separate *tac* promoters [22]. Expression of α-globin as a soluble fusion protein with exogenous heme has been attempted in bacteria, but expression and isolation proved to be more difficult than in the case of β-globin [23]. Vasseur-Godbillonn *et al.* [24] were able to improve the yield of soluble α-globin in *E. coli* by co-expressing the gene for the α-Hb stabilizing protein (AHSP), an erythroid-specific chaperone protein that binds specifically to free α-globin and prevents its precipitation [25], [26].

In the present report we describe modifications of expression conditions that increase the solubility of rHb and which also enhance the efficiency of post-translational modifications in *E. coli*. We identified optimal combinations of temperature, induction time, and expression strain for the efficient expression of soluble rHbs with proper cleaving of N-terminal methionine residues. The purified rHbs can be used for any number of downstream applications including structural and functional studies. Moreover, the protocol that we describe can be executed using resources of a standard molecular biology laboratory.

## Methods

### Study system

As a test case for our expression system, we produced rHbs of the deer mouse, *Peromyscus maniculatus*. Deer mice have served as subjects for extensive research on mechanisms of Hb adaptation to high-altitude hypoxia [27], [28], [29], [30], [31]. Highland and lowland populations of deer mice possess structurally distinct Hbs due to allelic variation at two tandemly duplicated α-globin genes and two tandemly duplicated β-globin genes. We refer to the high- and low-altitude α-globin sequences as Hα and Lα, respectively, and we refer to the high- and low-altitude β-globin sequences as Hβ and Lβ, respectively. With regard to the α-chain subunits, the Hα and Lα sequences differ at eight amino acid positions: 50(CD8)Pro/His, 57(E6)Gly/Ala, 60(E9)Ala/Gly, 64(E13)Gly/Asp, 71(E20)Ser/Gly, 113(GH1)His/Leu, 115(GH3)Ala/Ser, and 116(GH4)Glu/Asp (the notation in parentheses indicates the sequential number of each residue in α-helices A-H, the interhelical segments, or terminal extensions). With regard to the β-chain subunits, the Hβ and Lβ sequences differ at four amino acid positions: 62(E6)Gly/Ala, 72(E16)Gly/Ser, 128(H6)Ala/Ser, and 135(H13)Ala/Ser. To measure the net contributions of the α- and β-globin mutations to variation in Hb-O_2_ affinity, we synthesized the high- and low-altitude ‘wildtype’ isoHbs, Hα_2_Hβ_2_ and Lα_2_Lβ_2_, which we henceforth refer to as ‘HH’ and ‘LL’.

### Preparation of plasmid DNA

**Figure 1:**
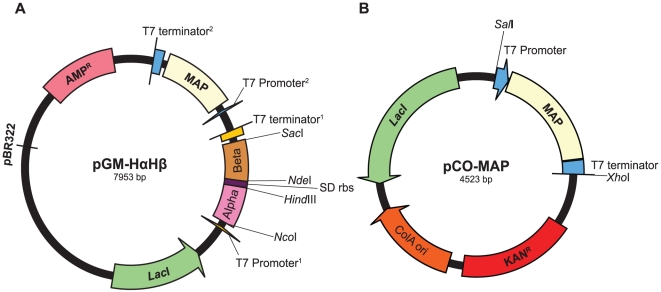
Design of the expression plasmids. As shown in Panel A, the pGM-HαHβ plasmid system contains a cassette of tandemly arrayed α- and β-globin genes with a Shine-Dalgarno ribosomal binding site as a spacer DNA. The MAP cassette is located downstream of β-globin. As shown in panel B, the pCO-MAP plasmid contains a MAP cassette under the control of a T7 promoter along with a kanamycin resistance gene.

We constructed two expression plasmids, pGM-HαHβ and pGM-LαLβ, which contain the α- and β-globin sequences that are charactistic of high- and low-altitude mice, respectively. The α/β-globin and MAP cassettes were synthesized by Genscript (Piscataway, NJ, USA). DNA sequences of the α- and β-globin genes were optimized with respect to *E. coli* codon preferences in order to maximize translational efficiency [23], [32]. The α- and β-globin genes were tandemly cloned with a Shine-Dalgarno ribosomal binding site as an intergenic spacer [19]. In addition to expressing the α- and β-globin genes, the pGM plasmid also expresses the MAP gene under the control of a second independent T7 promoter (**Figure 1a**). The MAP enzyme is responsible for cleaving the N-terminal methionine residues from the nascent globin chains, a critical post-translational modification of tetrameric Hb. We also co-expressed a pCO-MAP plasmid that contained an additional copy of the MAP gene with kanamycin resistance to provide a means of antibiotic selection (**Figure 1b**). Alternative α-globin genes can be swapped by using two unique restriction enzymes *Nco*I and *Hind*III, and alternative β-globin genes can be swapped by using a different pair of unique restriction enzymes, *Nde*I and *Sac*I.

### Optimization of expression conditions

Using the deer mouse pGM-HαHβ construct as a test case, we sought to increase the solubility of rHb by optimizing the expression conditions. Specifically, we conducted experiments to assess the combined effects of temperature, induction time, *E. coli* expression strain to enhance solubility and to improve the efficiencies of heme incorporation and post-translational modifications of deer mouse rHbs. The pGM-HαHβ plasmid was transformed into five commercially available expression strains: JM109 (DE3), BL21 (DE3) pLysS, BL21Star™ (DE3), BLR (DE3), and Origami™ (DE3). The *E. coli* cells were induced with 0.2 mM IPTG for 4 hr at 30°C in an orbital shaker at 200 rpm. The rHb samples were subsequently analyzed by 16% SDS-PAGE and yields were quantified by densitometric measures using the program Quantity One (BioRad, Hercules, CA, USA). We also evaluated four different combinations of temperature and induction time for the optimization of rHb expression: (1) 12°C for 24 hr, (2) 25°C for 16 hr, (3) 30°C for 4 hr, and (4) 37°C for 4 hr. As before, the samples were analyzed by 16% SDS-PAGE and yields were quantified by densitometric measures of band intensities. To assess whether the N-terminal methionine residues were properly cleaved, N-terminal peptide sequences of the α- and β-chain subunits were determined by means of Edman degradation. To prepare samples for peptide sequencing, the α- and β-globin monomers were separated by means of Acid-Urea-Triton (AUT) polyacrylamide gel electrophoresis [33], and were then transferred to PVDF membrane. The efficacy of the MAP enzyme in cleaving the N-terminal methionine residues was optimized by systematically altering the induction temperature, the induction time, and *E. coli* expression strain.

### Large Scale rHb expression

The pGM-HαHβ and pGM-LαLβ plasmids were initially transformed into the *E. coli* strain JM109 [34]. Based on results of our optimization experiments (see Results), the plasmid vectors were subsequently transformed into the JM109 (DE3) cells for large-scale production of rHbs. The pCO-MAP expression plasmid was co-transformed with the pGM-HαHβ and pGM-LαLβ plasmids. The *E. coli* cells were grown overnight in 2xYT medium with supplemented ampicillin (100 µg/ml) and kanamycin (50 µg/ml) at 37°C in an orbital shaker at 200 rpm. A 5 ml inoculum of the overnight culture was added to 250 ml of fresh TB medium with a final volume of 50 µg/ml ampicillin and kanamycin in a 1000 ml conical flask. Production was scaled-up in batches of 4 to 6 flasks containing 1–1.5 liter of TB medium. The cells were grown at 37°C in an orbital shaker at 200 rpm until the absorbance reached 0.6–0.8 at 600 nm. The cells were induced with 0.2 mM IPTG and were then supplemented with hemin (50 µg/ml) and glucose (20 g/L). The cells were then subsequently grown at 28°C for 16 hr in an orbital shaker at 200 rpm.

### Purification of rHb

**Figure 2:**
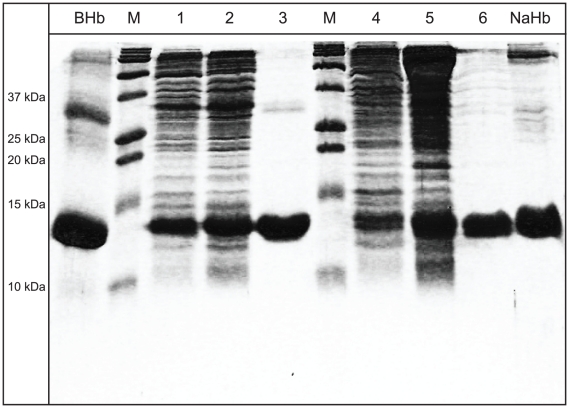
SDS-PAGE (20%) image showing the purified rHb isoforms. Lane BHb, Bovine Hb standard; Lane M, BioRad size standards; Lane 1, HH crude lysate; Lane 2, HH clarified fraction after dialysis; Lane 3, HH purified fraction; Lane 4, LL crude lysate; Lane 5, LL clarified fraction after dialysis; Lane 6, LL purified fraction; Lane NaHb, Native Hb from deer mouse blood.

The induced culture was saturated with CO for 15 min and the bacterial cells were harvested by centrifugation and stored frozen at −80°C. The cells were resuspended in lysis buffer (3 ml/g of cells), consisting of 50 mM Tris base, 1 mM EDTA, 0.5 mM DTT, and 1 mM PMSF. Lysozyme (1 mg/g of cells) was added along with the lysis buffer prior to sonication. The *E. coli* cells were sonicated with 10 sec pulse and a 20 sec pause on ice bath with 3.0 output duty for 15 min. Nucleic acids were precipitated by adding polyethyleneimine solution to a final concentration of 0.5 to 1%. The crude lysates were centrifuged at 15,000 g for 45 min at 4°C, and the clarified supernatants were then dialyzed overnight against CO saturated Tris-TETA buffer (20 mM Tris-HCl and 0.1 mM triethylenetetraamine, pH 7.4). The dialysed samples were centrifuged at 15,000 g for 30 min at 4°C and the supernatants were used for further purification. Recombinant Hbs were purified by means of HPLC using Q-Sepharose (anion-exchange) followed by SP-Sepharose (cation-exchange) pre-packed columns. The Q-Sepharose column was equilibrated with 20 mM Tris-HCl and 0.1 mM triethylenetetraamine, pH 8.3, and the bound rHbs were eluted by a linear gradient of 0 to 160 mM NaCl in Tris-TETA buffer pH 8.3. The eluted fractions were dialyzed with 10 mM sodium phosphate buffer at pH 7.2 and were concentrated with YM-30 centrifuge filter. The concentrated fractions were passed through SP-Sepharose column equilibrated with 10 mM sodium phosphate buffer at pH 7.2. The bound rHbs were eluted with a linear gradient of equilibration buffer versus 10 mM sodium phosphate buffer at pH 8.0 and concentrated with ultra filtration and stored at −80°C in aliquots with ∼0.5–1 mM heme. The qualities of the purified rHb samples were analyzed by 20% SDS-PAGE (**Figure 2**). Absorbance of the collected fractions was measured at 415 nm in a spectrophotometer (Beckman Coulter).

### Functional analysis of rHbs

Using the purified rHb samples, absorbance spectra of oxy, deoxy (obtained by adding a small amount of solid dithionite), and CO derivatives were measured at 450–600 nm to confirm that the absorbance maxima corresponded to those of native Hb. O_2_-binding equilibria of rHb solutions (0.2 mM heme) were measured at 37°C and in 0.1 M Hepes, pH 7.4, in the absence (*stripped*) and presence of added cofactors (0.1 M KCl^−^, and/or 2,3-diphosphoglycerate [DPG] at 2.0-fold molar excess over rHb tetramers [29]) as previously described [35], [36]. The measurements were conducted by using a modified diffusion chamber where changes in absorbance are recorded during stepwise changes in the O_2_ tension of equilibration gases. Experimental measures of P_50_ (O_2_ tension at half-saturation) and of the cooperativity coefficient n_50_ were obtained from the zero-intercept and the slope of Hill plots (log(Y/(1−Y)) vs. logPO_2_, respectively, where Y is the fractional O_2_ saturation.

## Results and Discussion

We initially tried to optimize the deer mouse rHbs using the protocol of Shen *et al.* [22]. In contrast to results obtained with human Hb (HbA), the deer mouse Hbs were expressed mostly in the insoluble fraction. Tetramers that incorporated the Hα subunit were characterized by an especially low solubility. The observed solubilities of the two deer mouse rHbs were consistent with *in silico* predictions using the method of Wilkinson and Harrison [37]. Using this same *in silico* method, we found that α- and β-chain Hbs from a diverse array of vertebrate species span a broad range of predicted solubilities (**Table S1**). These results suggest that obtaining adequate yields of soluble rHb may represent a chief obstacle to the experimental study of rHbs from many non-human animals.

**Figure 3:**
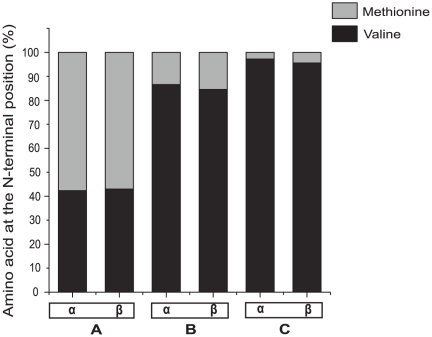
Variation in the efficiency of MAP enzyme in cleaving N-terminal methionine residues from the α- and β-chain subunits of purified HH rHb. The relative fractions of methionine and valine in the N-terminal residue positions are shown for three experimental conditions: (A) BL21Star™ (DE3) at 12°C for 24 hr, (B) BL21Star™ (DE3) at 30°C for 4 hr with co-expression of pCO-MAP expression plasmid, and (C) JM109 (DE3) at 28°C for 16 hr with co-expression of pCO-MAP expression plasmid. The efficiency of the MAP enzyme in cleaving the N-terminal methione residues from the α- and β-chain subunits is indicated by the relative fraction of methionine vs. valine in the N-terminal residue position. When the percentage of globin chains with NA1 valine exceeds 95%, this indicates that the post-translational modification has been carried out properly.

Our experimental results indicate that the yield of soluble rHb and the efficiency of heme incorporation vary among different *E. coli* strains. The selection of the right expression strain can greatly enhance the quality and quantity of rHb production. Of the five *E. coli* expression strains that we tested, BL21Star™ (DE3) produced the highest yield of soluble rHb (**Figure S1 a and S1 b**). Yields can be further enhanced by using the right combination of induction temperature and induction time. Using the pGM-HαHβ plasmid in the BL21Star™ (DE3) expression strain, we found that the highest yield of soluble rHb – relative to the insoluble fraction - was produced at low temperature with an extended induction time (12°C for 24 hr; **Figure S1 c** and **S1 d**). When supplemented with a rich medium, *E. coli* can continue to maintain a balanced growth in temperatures ranging from 10 to 49°C [38]. At low temperature, cold shock proteins such as CsdA, RbfA and CspA will be induced and they play important roles in protein synthesis [39], [40], [41]. Our preliminary results suggest that induction of the BL21Star™ (DE3) expression strain at 12°C for 24 hr yields good quantity of rHbs in the soluble fraction. We also assessed whether the N-terminal methionine residues were properly cleaved from the α- and β-chain subunits of the rHbs. To do this, the recombinant globin chains were separated by means of AUT gel electrophoresis and were then subjected to N-terminal peptide sequencing. We established an optimal culture condition to enhance the MAP activity without affecting the solubility of recombinant globin chains. We compared three combinations of induction temperature, induction time and expression strains: A) BL21Star™ (DE3) at 12°C for 24 hr, B) BL21Star™ (DE3) at 30°C for 4 hr with co-expression of pCO-MAP expression plasmid, C) JM109 (DE3) at 28°C for 16 hr with co-expression of pCO-MAP expression plasmid. The N-terminal peptide sequencing revealed that the rHbs expressed in JM109 (DE3) at 28°C for 16 hr with an additional copy of the MAP gene had the lowest fraction of intact N-terminal methionine residues in both the α- and β-chains (i.e., the post-translational processing was the most efficient) (**Figure 3**). Our results also revealed that the JM109 (DE3) strain generally produces rHb with a higher efficiency of heme incorporation compared to the BL21Star™ (DE3) strain. In general, an increased induction temperature enhances MAP enzyme activity but decreases the solubility of recombinant globin chains. Thus, the optimal expression conditions strike a balance between protein yield and proper post-translational processing.

**Figure 4:**
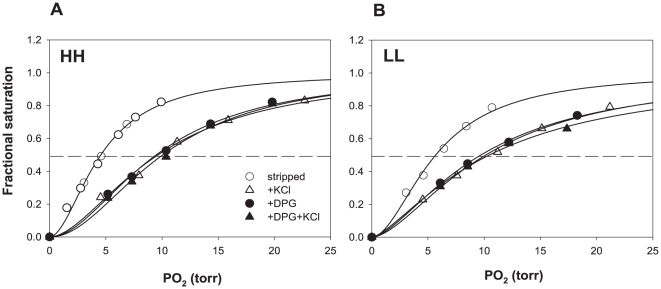
O_2_-binding properties of purified deer mouse rHbs. Panels A and B show O_2_ equilibrium curves for purified rHbs HH and LL, respectively. The rHbs HH and LL were expressed in the JM109 (DE3) *E. coli* strain at 28°C for 16 hr with co-expression of the pCO-MAP plasmid. O_2_-binding measurements were conducted at pH 7.4 at 37°C in presence and absence of allosteric cofactors (0.1 M Cl^−^, 0.1 M Hepes, 2.0-fold ratio DPG /rHb tetramers and 0.2 mM heme). Horizontal lines denote the half-saturation value for each rHb.

Relative to the LL rHb, the O_2_-binding experiments revealed that the HH rHb has a higher intrinsic O_2_ affinity (i.e., lower P_50(stripped)_) under the buffer conditions investigated (**Figure 4**) but similar affinity in the presence of anionic cofactors. The measured difference in intrinsic O_2_-affinity between the HH and LL rHbs is slightly larger than the measured O_2_-affinity differences between stripped red cell lysates from high- and low-altitude deer mice [30], [31]. The stripped HH and LL rHbs exhibited P_50_ values of 4.5 and 5.7 Torr and n_50_ values of 1.8 and 2.1, respectively (37°C, 0.1 M Hepes buffer, pH 7.4, heme concentration = 0.2 mM). Under identical experimental conditions, the stripped red cell lysates of high- and low-altitude mice exhibited mean P_50_ values of 7.4 and 7.9 Torr and mean n_50_ values of 2.1 and 2.3, respectively [31]. Individual deer mice generally express at least 3–4 distinct Hb components that contain different combinations of amino acid mutations in both the α- and β-chain subunits [30], [31], but the most common native Hb types in mice from high- and low-altitude have α- and β-chain primary structures that are similar (or identical) to those of the HH and LL rHbs, respectively. Although the red cells of high-altitude deer mice contain a preponderance of Hb components that are highly similar to the HH rHb, such components are typically co-expressed with other α- and/or β-chain isoforms that bear closer similarities to the LL rHb. Given the heterogeneous mix of distinct Hb types in the red cells of deer mice, we would not expect an exact match between P_50_ values for the composite hemolysates and those of isolated Hb components. Nonetheless, as expected, we do see a clear correspondence in the direction and magnitude of O_2_ affinity differences in the comparison between HH and LL rHbs and in the comparison between red cell lysates from high- and low-altitude mice. Because of the high level of Hb heterogeneity in the red cells of deer mice, it is clear that site-directed mutagenesis experiments involving recombinantly expressed Hbs will provide the most powerful means of identifying the specific amino acid mutations that are responsible for the observed differences in intrinsic O_2_ affinity between the high- and low-altitude Hb variants.

In summary, we identified combinations of expression conditions that can be expected to improve the qualitative yield of rHbs with low intrinsic solubilities in *E. coli*. This protocol should prove useful for the experimental study of rHbs in many non-human animals. Previous methods for expressing rHbs in *E. coli* involved the independent production of α- and β-chain subunits, followed by a second step to ensure proper heme incorporation during the assembly of the α_2_β_2_ tetramer. One of the chief advantages of our protocol is that we can express soluble rHb without co-expressing molecular chaperones like AHSP, and without the need for additional reconstitution or heme-incorporation steps. Moreover, our plasmid construct contains a combination of four unique restriction enzyme cut-sites flanking the α- and β-globin genes, which facilitates the swapping of alternative α- and β-globin variants to produce different tetrameric combinations.

## Supporting Information

Figure S1 **Optimization of HH rHb expression in** ***E. coli*** **.** (a) 16% SDS-PAGE image showing the expression profile of HH rHbs in different *E. coli* strains. Lane M, BioRad size standards; Lanes SF and ISF correspond to the soluble and insoluble fraction of rHbs in each of five different *E. coli* strains: (A) JM109 (DE3), (B) BL21 (DE3) pLysS, (C) BL21Star™ (DE3), (D) BLR (DE3), (E) Origami™ (DE3); Lane BHb, Bovine Hb standard; (b) Densitometric analysis of rHbs in the soluble and insoluble fractions in different *E. coli* strains. (c) 16% SDS-PAGE image showing the expression profile of HH rHbs at different conditions. Lane M, BioRad size standards; Lanes SF and ISF correspond to the soluble and insoluble fraction of rHbs under four different expression conditions: (1) 12°C for 24 hr, (2) 25°C for 16 hr, (3) 30°C for 4 hr, (4) 37°C for 4 hr; Lane BHb, Bovine Hb standard. (d) Densitometric analysis of rHbs at different expression conditions. Graph shows the soluble versus insoluble fraction ratios on the y axis, different expression conditions on the x axis.
(EPS)

Table S1 **Table showing** ***in silco*** **predictions of the intrinsic solubilities of hemoglobins from a phylogenetically diverse set of vertebrate species.**
(DOC)
