# Supplementary material for: Expression and Purification of Recombinant Hemoglobin in Escherichia coli
Source: PLoS One. 2011 May 20;6(5):e20176. doi: 10.1371/journal.pone.0020176 (PMC3098879; doi:10.1371/journal.pone.0020176)
Supplement: Table S1 — Table showing in silco predictions of the intrinsic solubilities of hemoglobins from a phylogenetically diverse set of vertebrate species. (DOC) [file pone.0020176.s002.doc]

| Species and common name | Alpha globin | | | Beta globin | | | Tetrameric HB |
| --- | --- | --- | --- | --- | --- | --- | --- |
|  | Accession number | Amino acids | Predicted Chance of insolubility % | Accession number | Amino acids | Predicted Chance of insolubility % | Predicted Chances of solubility in *E. coli* cells % |
| *Homo sapiens* (Human) | P01922 | 141 | 72.9 | P68871 | 146 | 61.6 | 32.75 |
| *Macaca mulatta* (Rhesus monkey) | P63108 | 141 | 77.1 | P02026 | 146 | 64.1 | 29.4 |
| *Oryctolagus cuniculus* (Rabbit) | P01948 | 141 | 73.1 | CAA24251 | 146 | 74.8 | 26.05 |
| *Rattus norvegicus* (Rat) | P01946 | 141 | 70.9 | CAA43137 | 146 | 53.7 | 37.7 |
| *Talpa europaea* (European mole) | P01951 | 141 | 81.9 | P02061 | 146 | 81.9 | 18.1 |
| *Cavia porcellus* (Guinea pig) | P01947 | 141 | 66.2 | P02095 | 146 | 66.1 | 33.85 |
| *Ursus maritimus* (Polar bear) | P68235 | 141 | 75.2 | P68011 | 146 | 74.8 | 25 |
| *Canis lupus familiaris* (Dog) | P60529 | 141 | 68.6 | P60524 | 146 | 72.8 | 29.3 |
| *Equus caballus* (Horse) | P01958 | 141 | 80.9 | NP_001157490 | 146 | 59.3 | 29.9 |
| *Ceratotherium simum* (Rhinoceros) | P01963 | 141 | 70.9 | P02066 | 146 | 53.6 | 37.75 |
| *Bos taurus* (Cow) | P01966 | 141 | 73.1 | AAA30408 | 144 | 57.2 | 34.85 |
| *Sus scrofa* (Pig) | P01965 | 141 | 77.1 | CAA60490 | 146 | 68.6 | 27.15 |
| *Hippopotamus amphibius* (Hippopotamus) | P19015 | 141 | 79.0 | P19016 | 146 | 55.9 | 32.55 |
| *Tursiops truncatus* (Dolphin) | P18978 | 141 | 80.9 | P18990 | 146 | 53.7 | 32.7 |
| *Loxodonta africana* (African elephant) | P01955 | 141 | 66.6 | P02085 | 146 | 50.7 | 41.35 |
| *Macropus giganteus* (Grey kangaroo) | P01975 | 141 | 58.7 | P02106 | 146 | 53.4 | 43.95 |
| *Ornithorhynchus anatinus* (Platypus) | P01979 | 141 | 51.0 | P02111 | 146 | 83.7 | 32.65 |
| **Peromyscus maniculatus* (Deer mouse) ^H^ | ABN71050 | 141 | 70.9 | ABY54987 | 146 | 68.4 | 30.35 |
| **Peromyscus maniculatus* (Deer mouse) ^L^ | ABN71058 | 141 | 64.0 | ACS16170 | 146 | 70.6 | 32.7 |
| *Psittacula krameri* (Parakeet) | P19831 | 141 | 77.1 | P21668 | 146 | 74.5 | 24.2 |
| *Gallus gallus* (Chicken) | P01994 | 141 | 63.5 | P02112 | 146 | 70.3 | 33.1 |
| *Struthio camelus* (Ostrich) | P01981 | 141 | 68.4 | P02123 | 146 | 70.3 | 30.65 |
| *Alligator mississippiensis* (Alligator) | P01999 | 141 | 61.4 | P02130 | 146 | 61.8 | 38.4 |
| *Xenopus laevis* (African clawed frog) | P02013 | 141 | 84.0 | P02133 | 146 | 83.5 | 16.25 |
| *Lepidosiren paradoxus* (Lungfish) | P02020 | 141 | 54.1 | P02138 | 147 | 77.9 | 34.0 |
| *Squalus acanthias* (Spiny dogfish) | P07408 | 141 | 53.1 | P07409 | 142 | 65.0 | 40.95 |
| *Dasyatis akajei* (Stingray) | P56691 | 141 | 66.9 | P56692 | 141 | 55.2 | 38.95 |
